# Selective symmetrical necrotizing encephalopathy secondary to primary mitochondrial disorder in a cat

**DOI:** 10.1111/jvim.16222

**Published:** 2021-07-22

**Authors:** Elena Dell'Era, Margherita Polidori, Marco Bernardini, Stefano Capomaccio, Katia Cappelli, Federica Balducci, Maria T. Mandara

**Affiliations:** ^1^ Neurology Unit AniCura Portoni Rossi Veterinary Hospital Bologna Italy; ^2^ Department of Veterinary Medicine, Neuropathology Laboratory University of Perugia Perugia Italy; ^3^ Department of Animal Medicine, Production and Health, Clinical Section University of Padua Legnaro Italy; ^4^ Department of Veterinary Medicine, Laboratory of Molecular Biology University of Perugia Perugia Italy

**Keywords:** cat, magnetic resonance imaging, mitochondriopathy, necrotizing encephalopathy, tRNA‐Leu^(UUR)^ gene

## Abstract

A 2‐year‐old female cat was referred for progressive neurological signs indicative of involvement of the prosencephalon, cerebellum, and brainstem. Magnetic resonance imaging identified multifocal, bilateral, symmetrical lesions with strong contrast enhancement, affecting multiple areas of the brain. Neuropathology at necropsy showed demyelination, necrotic lesions, spongiosis, and neuropil edema with reactive astrogliosis and neovascularization. Ultrastructural study indicated mitochondrial polymorphism. Genetic investigations outlined 2 polymorphisms within the tRNA‐Leu^(UUR)^ gene of mitochondrial DNA. Imaging and neuropathological findings were consistent with selective symmetrical necrotizing encephalopathy, for which genetic investigations support mitochondrial pathogenesis.

**Abbreviations**
AHE: Alaskan Husky encephalopathy
CSF: cerebrospinal fluid
DWI: diffusion weighted imaging
FFPE: formalin‐fixed paraffin‐embedded
FLAIR: fluid‐attenuated inversion recovery
LFB‐PAS: luxol fast blue‐periodic acid Schiff
LSD: lysosomal storage disease
MELAS: mitochondrial encephalopathy, lactic acidosis, and stroke‐like episodes
MRI: magnetic resonance imaging
nDNA: nuclear DNA
PMD: primary mitochondrial disorder
SSNE: selective symmetrical necrotizing encephalopathy
TEM: transmission electron microscopy

## 1 CASE DESCRIPTION AND CLINICAL FINDINGS

A 2‐year‐old female spayed domestic shorthair cat was presented with a complex history of movement deficits, staggering, difficulty jumping, daily episodes of obtundation and disorientation, and progressive generalized rigidity. The cat was adopted from a rescue center at the presumed age of 3 months. The cat lived indoors and was fed commercial pet food.

Neurological signs appeared shortly after adoption, with subtle onset and slow progression over the next 18 months.

At the time of presentation, the cat had an extremely obtunded mental status. Subtle vestibular ataxia was present; postural reactions were mildly decreased in all 4 limbs. Menace response and vestibulo‐ocular reflex were severely decreased bilaterally. Positional ventral strabismus also was present bilaterally. The remainder of the neurological examination was normal.

On the basis of the neurological findings, intracranial, multifocal lesions were suspected. Considering the chronic and slowly progressive nature of the clinical signs and the multifocal localization, degenerative, metabolic, or congenital disorders were considered the most likely differential diagnoses.

**FIGURE 1:**
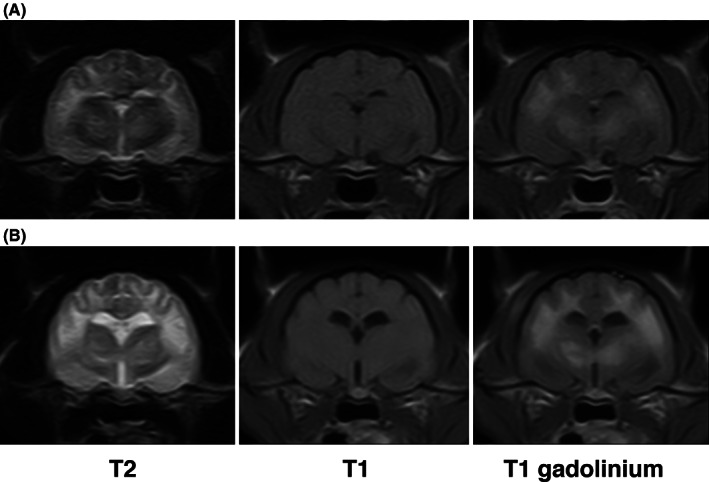
Transverse magnetic resonance images of the brain at the level of the thalamus in two studies performed 6 months apart. In the initial images (A), there is a diffuse, almost symmetrical, hyperintensity in T2‐weighted images affecting the white matter of both hemispheres and both thalami. These areas are isointense in T1‐weighted images and enhance slightly after contrast medium administration. In the second MRI study (B), the hyperintensity in T2‐weighted images is more pronounced as well as the contrast. MRI, magnetic resonance imaging

A CBC and serum biochemistry profile were unremarkable. The cat underwent low field magnetic resonance imaging (MRI) (MrJ, 0.22 T, Paramed, Genua, Italy) of the brain under general anesthesia. The MRI study included fast spin echo T2‐weighted sequences (T2W; repetition time, 3000‐3670 ms; echo time, 120 ms; slice thickness, 3.5‐4 mm), spin echo T1‐weighted sequences (T1W; repetition time, 500‐687 ms; echo time, 24‐26 ms; slice thickness, 3.5‐4 mm) pre‐ and post‐IV injection of contrast medium (gadoteric acid, 0.1 mmol/kg; Dotarem, Guerbet, Italy), and fluid‐attenuated inversion recovery sequences (FLAIR; repetition time, 4633 ms; echo time, 90 ms; inversion time, 1150 ms; slice thickness, 3.5 mm). The MRI study showed marked hyperintensity with poorly defined margins on T2W and FLAIR images affecting the entire corona radiata in both hemispheres, more pronounced in the occipital lobe. Diffuse hyperintensity was identified on T2W and FLAIR images in the dorsal part of both cerebellar lobes, with poor distinction between white and gray matter. Symmetrical lesions with the same signal features were found in the thalamus and in both caudal colliculi. A unilateral lesion also was found in the right mesencephalon. All of the lesions were isointense on T1W imaging, most of them showing marked post contrast enhancement (Figure 1).

Cerebrospinal fluid (CSF) examination disclosed no abnormalities in protein concentration or cell count, but cytoplasmatic vacuolization with phagocytosed material was seen within macrophages. Based on the clinical history and imaging findings, macroscopic congenital defects were ruled out, whereas degenerative and metabolic diseases still were suspected, with storage disease as the primary differential diagnosis.

Clinical signs worsened in the 6 months after discharge before the cat underwent a second neurological examination, which showed deterioration of all of the previously identified neurological deficits. Because of poor prognosis, the owner elected euthanasia and consented to a follow‐up MRI study beforehand. The MRI study disclosed a mild increase in extension and stronger contrast uptake of the previously identified lesions. Furthermore, a new lesion in the pons with the same signal features as well as mild ventricular ectasia now was observed (Figure 1). Collection of CSF was not performed at this time.

## 2 NECROPSY FINDINGS

**FIGURE 2:**
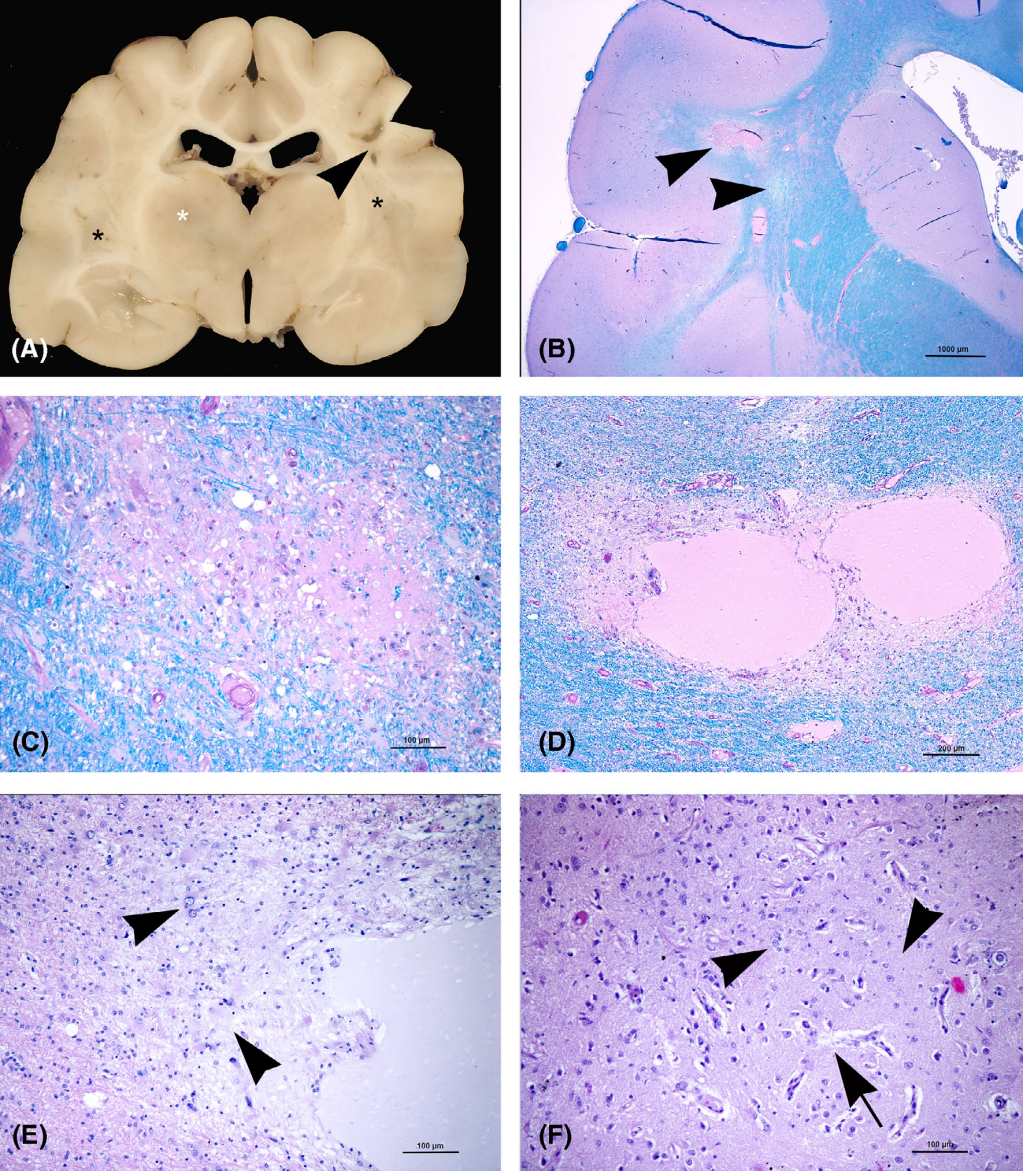
A, Gross lesions. Transverse brain section at level of thalamus. Bilateral areas of discoloration (black asterisks) and cystic lesions (arrowhead) involving the corona radiata. A discoloration area is also present in the thalamus (white asterisk). B‐F, Histological lesions. B, Transverse brain section at the level of nucleus caudatus. The corona radiata shows pale blue areas (arrowheads) consistent with demyelination (LFB‐PAS). C, Detail of (B). Periventricular white matter. Area of LFB discoloration with mild spongiosis (LFB‐PAS). D, Radiate white matter. Necrotic cystic lesions containing pale eosinophilic proteinaceous material (LFB‐PAS). E, Detail of (D), showing a number of reactive astrocytes (gemistocytes) (arrowheads) around the cystic lesion hematoxylin‐eosin (H&E). F, Nucleus caudatus. Increased density of microvessels (neovascularization) (arrow) associated with gliosis (arrowheads) hematoxylin‐eosin (H&E). LFB‐PAS, luxol fast blue‐periodic acid Schiff

**FIGURE 3:**
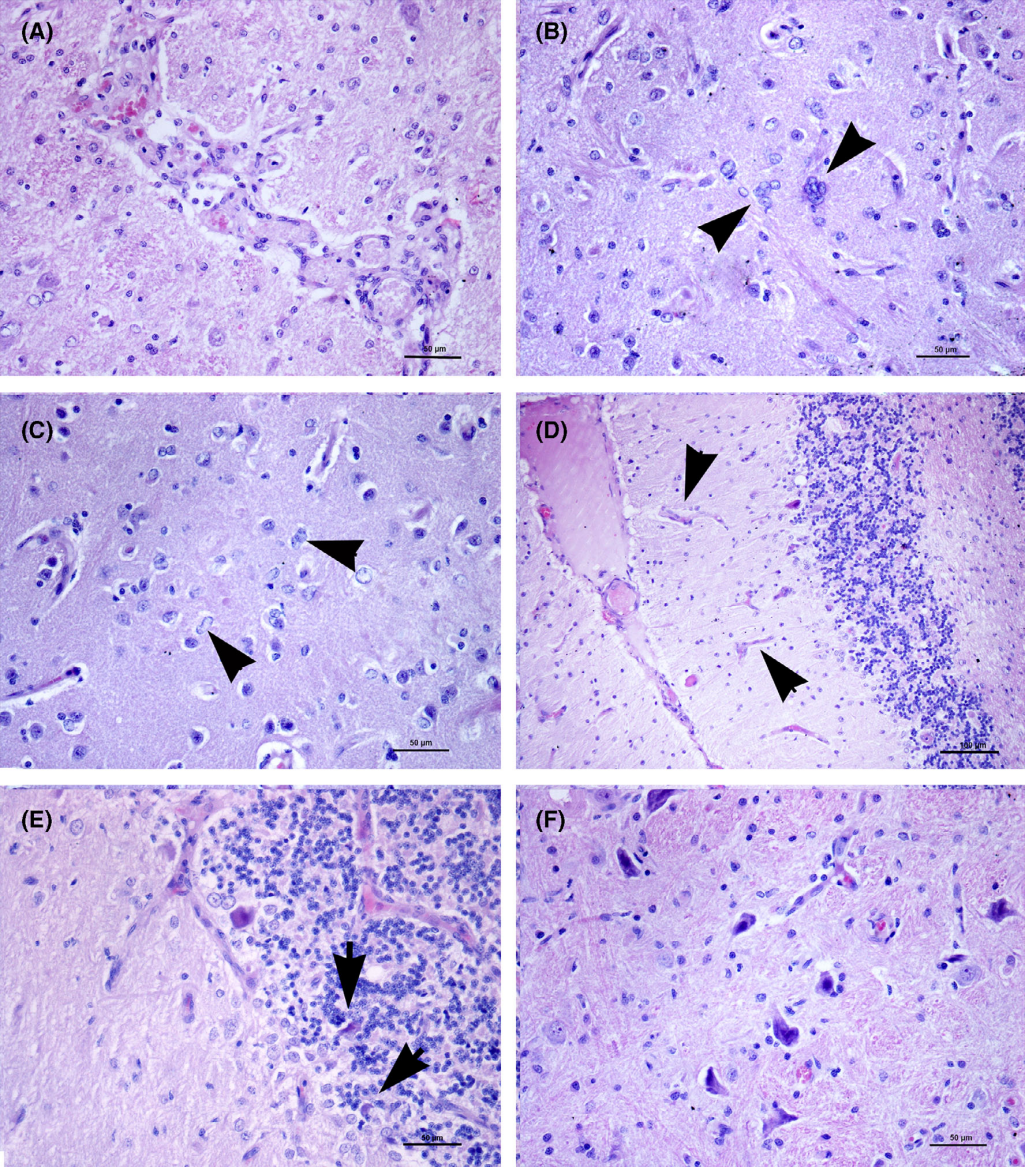
A‐F, Histological lesions. A, Nucleus caudatus. Marked proliferation of endothelial cells in the areas affected by neovascularization (HE). B, Occasional Alzheimer type I cells showing from 3 to 6 small nuclei (arrowheads) (HE). C, Focus of astrocytosis and neovascularization. A number of astrocytes show swollen or lobated nucleus (Alzheimer type II cells) (arrowheads) (HE). D, Cerebellar cortex. Areas of neovascularization in the molecular layer (arrowheads) hematoxylin‐eosin (H&E). E, Cerebellar cortex. In this folium, proliferation of Bergmann's astrocytes is associated to occasional shrunken Purkinje cells consistent with apoptotic bodies hematoxylin‐eosin (H&E). F, Medial vestibular nuclei. Neuronal basophilic inclusions consistent with calcification hematoxylin‐eosin (H&E)

Necropsy was performed with the owner's consent. Macroscopic examination of 0.5 cm formalin‐fixed transverse brain sections disclosed bilateral symmetrical small brownish areas in the caudate nuclei, thalamus, periaqueductal gray matter, vestibular nuclei, and subcerebellar brainstem. White matter associated with the right parietotemporal cortex had extended symmetrical discoloration with small cystic lesions (Figure 2A). Paraffin‐embedded hematoxylin‐eosin (H&E) stained 5 μm sections were prepared for routine histological examination. Selected specimens also were stained with luxol fast blue‐periodic acid Schiff (LFB‐PAS). Demyelination with associated spongiosis and marked LFB discoloration was seen in the corona radiata and the periventricular white matter (Figure 2B,C). In some areas, tissue rarefaction developed into malacic cystic lesions surrounded by marked gliosis characterized by gemistocytes and Alzheimer type II cells (Figure 2D,E). Occasional Alzheimer type I cells and neuronal calcifications were identified in the adjacent cerebral cortex. Neuropil edema also was detected in the adjacent cortex. Tissue rarefaction also was observed in the thalamus (caudal lateral nuclei and optic tracts) and mesencephalic reticular formation. Diffuse neovascularization with hypertrophic endothelial cells (Figures 2F and 3A) and astrocytosis with typical Alzheimer type I and type II cells (Figure 3B,C) were found within the head of the caudate nuclei, thalamus, perithalamic internal capsule, caudal crus, periaqueductal gray matter, and caudal colliculi. In the cerebellum, histological examination identified bilateral symmetrical foci of white matter discoloration and gliosis. Moreover, multifocal neovascularization in the molecular layer (Figure 3D) and occasional Purkinje cell apoptosis, associated with Bergmann's gliosis, were observed (Figure 3E). The lateral cerebellar nuclei showed an apparent depletion of neurons in a gliotic background. Necrotic sites also were detected in the medial vestibular nuclei associated with neuronal calcifications (Figure 3F). Selected formalin‐fixed paraffin‐embedded (FFPE) samples were submitted for immunohistochemical investigation. Antibodies against glial fibrillary acid protein for astrocyte identification (polyclonal‐rabbit, Dako, Code N° Z0334; dilution, 1 : 200) and MAC387 for macrophage identification (monoclonal‐mouse anti‐human, Dako, Code N° M0747; dilution, 1 : 250) were used. A thick glial fibrillary acid protein positive fibrillary network consistent with astrocytic gliosis was observed in the areas of tissue rarefaction and demyelination. The MAC387‐reaction was negative in all of the lesions, confirming the absence of a macrophage reaction.

**FIGURE 4:**
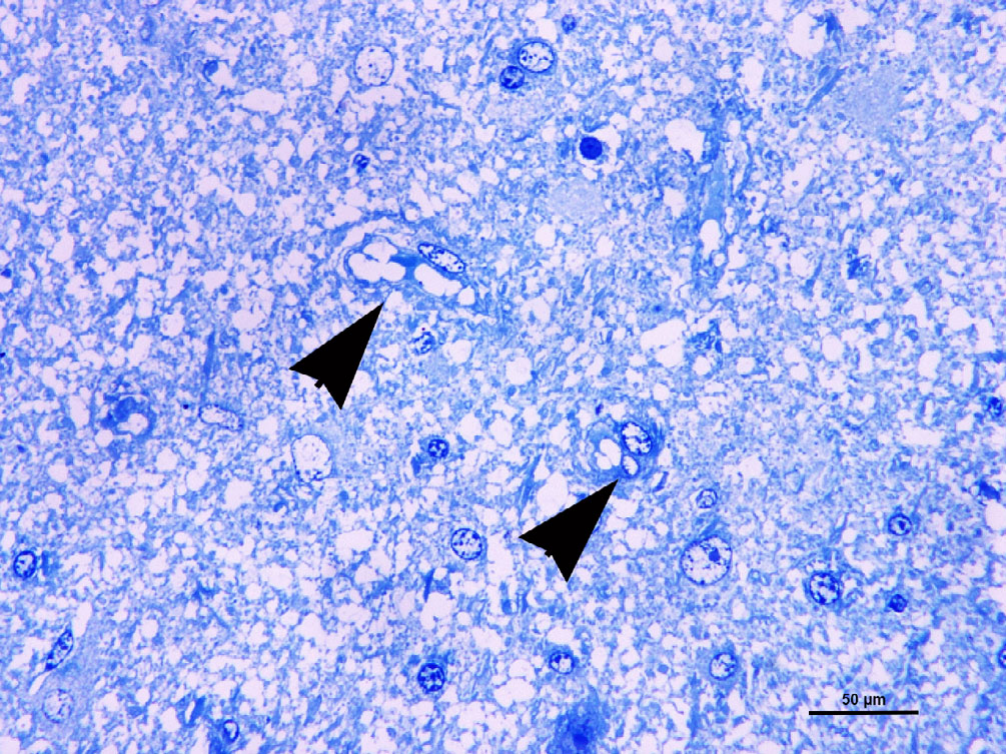
Cerebral white matter. Diffuse vacuolization of nervous tissue. Two astrocytes show cytoplasm vacuoles (arrowheads) (semithin section, toluidine blue)

Selected FFPE samples obtained from the discolored radiate white matter were submitted for transmission electron microscopy (TEM) following Craciun and Horbin's modified protocol.^1^ Semithin sections confirmed the presence of neuropil vacuolization along with endotheliocyte swelling, perivascular plasma effusion, Alzheimer cells, and vacuolated astrocytes (Figure 4). On TEM, mitochondrial diameters ranged from approximately 0.5 to 7 μm. Irregular mitochondrial crests, electron‐dense bodies, and myelin sheath splitting also were observed.

Extraction of DNA (kit MagneSil Genomic, Fixed Tissue System‐Promega) was followed by PCR amplification with customized primers and sequencing of the obtained amplicon.

**FIGURE 5:**
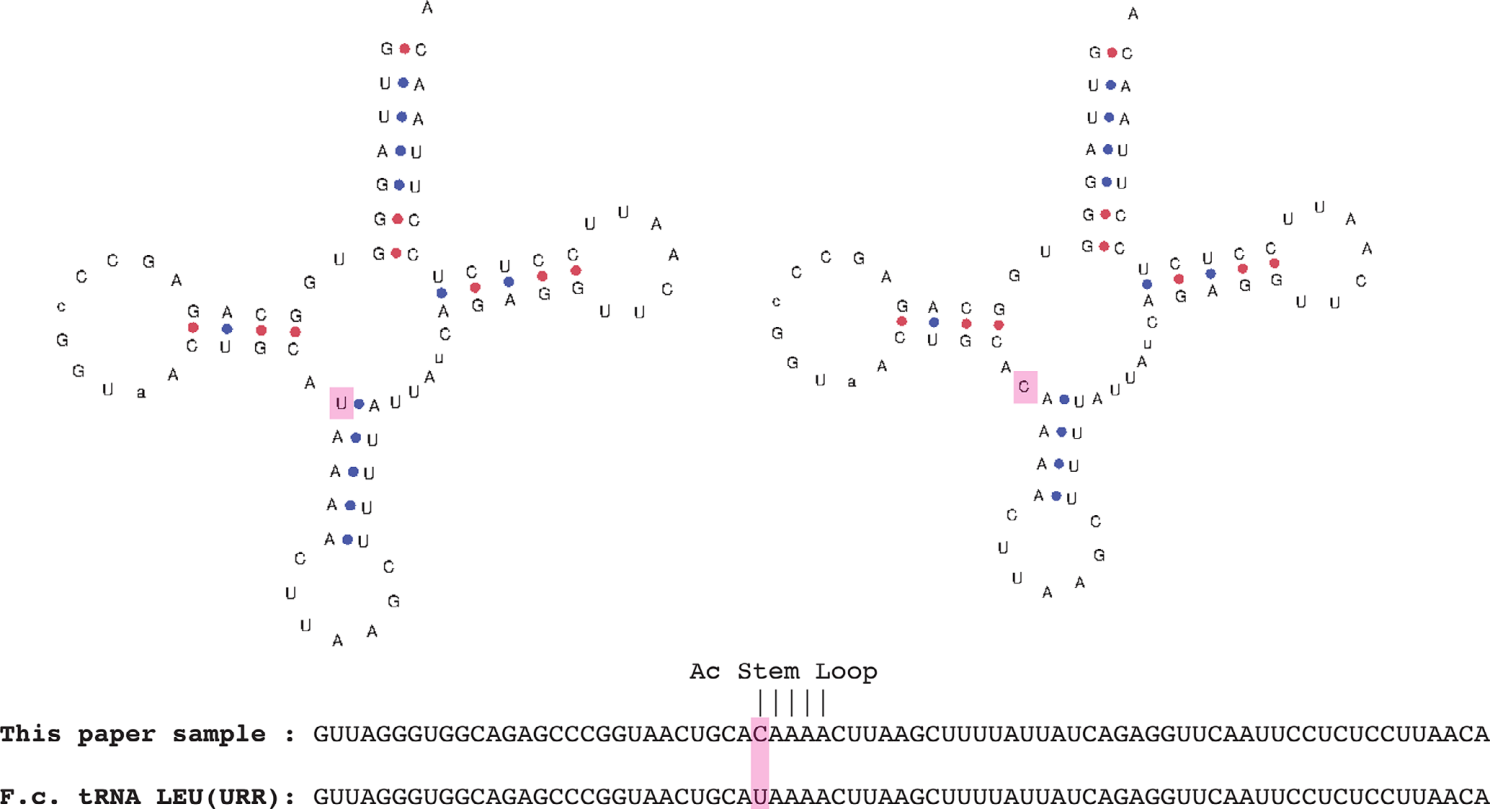
Contextualization of the anticodon stem loop mutation found in this study. On the left panel, the wild‐type 2D structure it tRNA‐Leu with intact alignment of the anticodon stem loop; on the right panel, the 2D structure calculated from our sample. On the bottom, the alignment of the two sequences. In pink is highlighted the mutation as well as its location in the structures. 2D structures were produced with tRNAscan‐SE36

Primers were designed based on mitochondrion reference sequence (NC_001700.1) spanning 260 bp containing the tRNA‐Leu^(UUR)^ gene (forward, 5′‐GTGAGGCCCACTTCACCAAA‐3′; reverse, 5′‐ GCCTAGCACTTTTCGTTCGAC‐3′). The PCR was performed using 30 ng of DNA as template for the amplification with the following protocol: 94°C for 3 minutes as initial denaturation, then 35 cycles of 94°C for 10 seconds, 58°C for 15 seconds, and 72°C for 30 seconds. The reverse and forward sequences obtained were aligned (Contig Express‐Vector NTI Suite 9, Invitrogen) and contig identified 2 polymorphisms with respect to the reference: (a) A‐to‐G transition at position 136 and (b) A‐to‐T transversion at position 189 of the PCR product. Both changes were present in a heteroplasmic fashion. The first 1 was located within the tRNA‐Leu^(UUR)^ gene at position 29 of the Ac‐stem in the mitochondrial *Felis catus* DNA (Figure 5).

Gross, histological, and electron microscopy findings were consistent with selective symmetrical necrotizing encephalopathy (SSNE) for which genetic investigations supported a mitochondrial pathogenesis.

## 3 DISCUSSION

Primary mitochondrial disorders (PMDs) are the expression of impaired mitochondrial activity caused by an inherited mutation in the organelle's DNA (mtDNA) or nuclear DNA (nDNA).^2^, ^3^ Spontaneous de novo mutations rarely are reported.^4^ Typically, PMDs are multisystemic disorders, with musculoskeletal and nervous tissues most severely affected because of their high energy demand.

In human medicine, the onset of PMDs shows a bimodal distribution. The first peak occurs in the first 3 years of life with severe, often fatal clinical signs caused by either an autosomal recessive nDNA mutation or mtDNA with high rate of heteroplasmy. The second peak is broader and frequently caused by mtDNA mutation manifesting itself in young people and adults with less severe clinical signs.^4^, ^5^, ^6^, ^7^, ^8^, ^9^

In vivo diagnosis of PMDs is challenging and no established protocols exist.^10^ Clinical presentation and diagnostic findings vary with the pathology but also among patients suffering from the same disease. Clinical signs are often vague and diagnosis requires at least clinical recognition of a metabolic disorder, MRI studies, and identification of the DNA mutation. Increased blood lactate concentration and lactate peak on magnetic resonance spectroscopy are additional findings indicating an energy impairment, but they are neither pathognomonic nor always present.

For decades, the only way to provide a suspected diagnosis of a PMD in animals consisted of recognizing neuropathological findings similar to those reported in cases of PMDs in humans.^11^ The first report focused on a polioencephalomyelopathy resembling Leigh's syndrome in 3 Australian cattle dog puppies.^12^ This report was followed by a number of reports in other breeds,^13^, ^14^, ^15^, ^16^, ^17^, ^18^, ^19^, ^20^, ^21^ with the Alaskan Husky being most commonly represented.^19^, ^20^, ^21^ Kittens affected by a suspected mitochondriopathy were identified once in 1979^22^ and reported in 1995.^23^ In humans, however, PMDs show great heterogeneity in clinical presentation and lesion distribution, and lack pathognomonic patterns.^10^ Therefore, where recognition of similarities with a specific PMD of humans remains necessary, today genetic sequencing plays a more important role in the final diagnosis.

In 2006, a mtDNA mutation was linked to an inherited spongiform leukoencephalomyelopathy reported in 2 families of Australian cattle dogs and Shetland sheepdogs impairing the activity of cytochrome b.^24^ In 2009, a neutral polymorphism in tRNA‐Leu^(UUR)^ of mtDNA was identified as an A‐G transition in Yorkshire terriers affected by Leigh‐like subacute necrotizing encephalopathy.^14^ In 2013, a mutation in the thiamine transporter 2 gene leading to Alaskan Husky encephalopathy (AHE) was described, making this disorder a secondary mitochondriopathy and not a PMD as previously suspected.^21^

The cat described here had a history suggestive of a progressive multifocal encephalopathic disorder. Abnormal mental status, decreased menace response and postural reactions in all 4 limbs, vestibular ataxia, slow vestibulo‐ocular reflex, and positional strabismus indicated involvement of both forebrain and brainstem, and possibly cerebellum.

The young age of onset and slow progression of clinical signs were suggestive of a degenerative disease or metabolic disorder. A congenital defect of brain development was considered less likely, because of the progressive clinical course.

Hepatic encephalopathy was considered a differential diagnosis for the slowly progressive neurological signs and poor clinical conditions. However, ptyalism was never observed, the neurological deficits were never related to feeding, and gastrointestinal signs were not reported. No clinically relevant changes were found on hematology and serum biochemistry, making metabolic disorders unlikely.^25^, ^26^

In veterinary neurology, the best known degenerative diseases are the lysosomal storage diseases (LSDs), which are characterized by progressive, multifocal, or diffuse encephalopathy.^27^ Abnormal behavior, ataxia, and proprioceptive deficits are common, but clinical presentations vary.

Lesion distribution on MRI could have been suggestive of LSDs and mildly supported by CSF analysis, which identified cytoplasmic vacuolization in macrophages. Therefore, LSD was considered the main differential diagnosis in this cat after the first consultation and remained so at the time of euthanasia. However, the strong contrast enhancement of the lesions observed throughout the brain is reported infrequently in the human or veterinary medical literature for these conditions.^28^

The brain MRI of our cat was characterized by a bilateral, almost symmetrical, pattern of iso‐ or hypo‐intense lesions on T1W sequences that appeared hyperintense on T2W and FLAIR sequences. This pattern is consistent with the brain MRI of PMD‐affected human patients and is compatible with astrogliosis, vasculopathy, and cystic malacic lesions^29^ observed at histopathology. In neuroimaging of humans, PMD‐related lesions show a more restricted diffusion in diffusion‐weighted imaging (DWI).^29^, ^30^, ^31^, ^32^ The usefulness of DWI to better investigate microstructural alteration in the brain of animals affected by PMDs is not yet defined. In our case, DWI sequences were not acquired because they are not available in a low‐field MRI system.

The most striking MRI feature in our cat was the strong contrast enhancement of the lesions. In the few previously published MRI descriptions of PMDs in veterinary medicine,^14^, ^15^, ^19^ no postcontrast enhancement has been reported. In MRI of the brain in PMD‐affected human patients, contrast enhancement rarely is described.^29^, ^30^, ^33^ When present, enhancement seems to be associated with vasculopathy and white matter rarefaction.^34^ In our case, we hypothesize that the strong contrast enhancement was caused by 2 factors: the diffuse involvement of white matter and the neovascularization with endothelial changes, causing increased contrast leakage in the brain. These changes also were observed on histopathology.

To reach a definitive diagnosis, the brain was submitted for neuropathology. Bilateral symmetrical malacia and demyelination were reported as the most characteristic findings. Transmission electron microscopy identified clinically relevant mitochondrial polymorphism, but caution should be exercised when interpreting TEM findings obtained using FFPE tissue samples. The identification of 2 point mutations in the tRNA‐Leu^(UUR)^ gene coding for mitochondrial tRNA‐Leu supported the diagnosis of PMD in the cat.

The reported neuropathology findings call for comparison with similar selective symmetrical necrotizing encephalopathies reported in human and veterinary medicine. If distribution of the brain lesions in this cat did not overlap any specific human or canine PMD pattern, involvement of cerebral white matter and cerebellar cortex resembles that observed in Pearson/Kerns‐Sayre syndrome (P/KSS) in humans,^29^ whereas involvement of the thalamus and brain stem is similar to what is observed in Leigh syndrome in humans,^8^ necrotizing AHE and subacute necrotizing encephalopathy in Yorkshire Terriers.^14^, ^20^ Indeed, compared to SSNE of these dog breeds, subcortical white matter lesions in our cat were much more severe whereas thalamic and brain stem lesions did not occur with a V‐shaped pattern.^14^ Similar to a previous study,^20^ we identified active degeneration in the caudate nuclei, thalamus, and brainstem, primarily characterized by gliosis and prominent blood vessel reactivity. Quiescent lesions, namely burned out lesions, instead were recognizable in the cerebral hemispheres based on advanced gemistocytic gliosis, spongiosis, and cavitary changes. Vascular proliferation is a common finding in humans^35^ and dogs with mitochondrial encephalopathies.^14^, ^20^ Marked reactive vascular hypertrophy and endothelial hyperplasia both suggest oxidative damage to the endothelial cells and nervous tissue, even when the latter is not yet evident.^36^

Polymerase chain reaction and genetic sequencing allowed us to identify 2 heteroplasmic polymorphisms in the DNA of our cat. One of them was located within the tRNA‐Leu^(UUR)^ gene, affecting the Ac Stem loop in the secondary leaf structure of mitochondrial tRNA‐Leu, which often is a pathogenic hotspot in humans and other species as well.^37^, ^38^ This mutation corresponds to the DNA sequence of the T3258C mutation detected in the MELAS syndrome of humans.^39^ Nevertheless, determining the pathogenicity of specific mutations in mitochondrial tRNA and predicting clinical outcomes appears to be extremely difficult.^37^ Additional studies to screen extensive sequence databases in the cat (*F catus*) are needed to establish causality between genotype and phenotype in this disease.

## 4 CONCLUSIONS

The PMDs are neurological disorders rarely encountered in veterinary medicine, and complete reports that support clinicians in making a diagnosis are lacking. They generally develop as a selective symmetrical necrotizing encephalopathy. For this reason, they must be considered as a differential diagnosis when degenerative diseases or metabolic disorders are suspected based on clinical examination, and multifocal, bilateral, symmetrical central nervous system lesions are found on MRI. Although medical treatment still is being investigated and prognosis remains guarded to poor, a complete diagnostic evaluation should be performed whenever possible. Future studies should aim to identify an animal model for studies of human disease and also to exchange information among disciplines to broaden the knowledge base for these neurodegenerative diseases.

## CONFLICT OF INTEREST DECLARATION

Authors declare no conflict of interest.

## OFF‐LABEL ANTIMICROBIAL DECLARATION

Authors declare no off‐label use of antimicrobials.

## INSTITUTIONAL ANIMAL CARE AND USE COMMITTEE (IACUC) OR OTHER APPROVAL DECLARATION

Authors declare no IACUC was needed. The cat's owner consented to all the postmortem investigations.

## HUMAN ETHICS APPROVAL DECLARATION

Authors declare human ethics approval was not needed for this study.
